# Appropriateness in allergic respiratory diseases health care in Italy: definitions and organizational aspects

**DOI:** 10.1186/s12948-016-0042-3

**Published:** 2016-04-19

**Authors:** Carlo Lombardi, Eleonora Savi, Maria Teresa Costantino, Enrico Heffler, Manlio Milanese, Giovanni Passalacqua, Giorgio Walter Canonica, Italian Allergic Respiratory Diseases Task Force

**Affiliations:** Departmental Unit of Allergology-Clinical Immunology and Respiratory Diseases, Fondazione Poliambulanza of Brescia, Via Bissolati, 57, Brescia, Italy; Departmental Unit of Allergology, AUSL of Piacenza, “Guglielmo da Saliceto” Hospital, Piacenza, Italy; Unit of Allergology, Department of Internal Medicine, Azienda Ospedaliera “Carlo Poma” of Mantua, Mantua, Italy; Respiratory Medicine and Allergy-Department Clinical and Experimental Medicine, University of Catania, Catania, Italy; Unit of Pulmonology, S. Corona Hospital, Pietra Ligure, Savona, Italy; Allergy and Respiratory Diseases, IRCCS San Martino-IST-University of Genoa, Genoa, Italy

**Keywords:** Allergology, Appropriateness, Slow medicine, Choosing wisely, Italy, National Health System, Health resources

## Abstract

In a historical period in which sustainability of the National Health Service is mandatory because of the international economical situation, the limited available resources at national level and the tendency of passing from a “population medicine” model towards the concept of “individualized medicine”, the debate on appropriateness of medical and surgical procedures is of central importance. The choosing wisely campaign, started in United States in 2012 and then spread all over the world, tries to summarize which are the most inappropriate procedures for each medical and surgical speciality; as far as allergic respiratory diseases, the most relevant Italian societies and the American Academy defined the allergological procedures with the highest probability of inappropriateness. In Italy, a recent decree of the Ministry of Health defined a list of more than 200 procedures that will be considered as inappropriate in certain conditions; many of these procedures concern allergology, including allergic respiratory diseases. In this commentary we discuss the above mentioned decree and the concept of appropriateness in the field of allergic respiratory diseases, trying to figure out some practical considerations based on the current health resources available in the field of allergology in Italy.

## Background

Appropriateness policies have the main goal to both contain health care spending and efficiently redistribute resources. In fact, the inappropriateness may be in excess (“overuse”) or in defect (“underuse”): the reduction of the first one recovers lost resources, and the implementation of the second one requires investments [1]. Therefore, any strategy to reduce the inappropriateness must be guided by the principle of “disinvestment and reallocation.” Today, a medical prescription can not take into account only the individuality of each patient (“individualized medicine”), but also the unacceptable variability in the requirements (“population medicine”) and the fact that every doctor is required to contribute, as manager of public resources, to sustainability of the National Health Service. Until a few decades ago, the work of the clinicians did not pose the question of the appropriateness so urgently as today: the aim of the physician at the bedside appeared unique, the tools to achieve it were few and the simple medical evaluation was more than enough. Current clinical practice instead it is a complex activity that involves different purposes, therefore taking into account many heterogeneous elements. Appropriateness is often treated as a single concept. However, there are two distinct types of appropriateness: “professional” appropriateness, closely linked to the cultural level of the health care and decision-making, and appropriateness of the setting in which care is provided [2]. It is therefore clear that these two distinct levels of appropriateness must necessarily be synergistic and not discordant. Also, appropriateness cannot be separated from other essential concepts: efficacy, effectiveness and cost-effectiveness. When quality of healthcare is the matter of Healthcare, as universally agreed, quality must be effective, efficient, safe, humanized and consider the economic resources [3]. Furthermore, it is equally essential that the appropriateness is applied within the concept of evidence-based medicine (EBM) [4]. Finally, appropriateness should always be linked to avoidance of over diagnosis malpractice [5]. A shift in dynamics of diagnostic medicine to behaviors of appropriateness also well correlates with the new cultural movements of “choosing wisely” and “slow medicine”. choosing wisely is an initiative launched by the ABIM Foundation and then spread all over the world. choosing wisely aims to promote the interaction between clinicians and patients in order to support patients in choosing care that is supported by evidence, not duplicative of other tests or procedures already received, free from harm, and truly necessary [6–9].

**Fig. 1:**
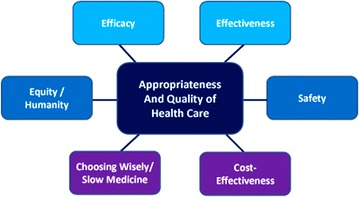
Fundamental components and objectives to improve the appropriateness and the quality of health care

Choosing wisely recommendations meant to identify which are appropriate and necessary procedures or treatments. As each patient situation is unique, providers and patients should use the recommendations as guidelines to determine an appropriate treatment plan together. In other words, a diagnostic or therapeutic procedure can be considered appropriate when it is delivered according to clinical indications with proven efficacy, at the right time, in accordance with appropriate mode. In Italy, the appropriateness of healthcare is increasingly gaining the interest health care providers and policy-makers. In fact, in Italy, a major topic of the current health care debate is that a substantial proportion of the health care delivered is inappropriate. Recently, the Italian Ministry of Health has presented a decree, known as “Appropriateness Decree”. This decree has been published in the “Gazzetta Ufficiale” of January 20, 2016, and it contains a list of more than 203 diagnostic tests with the criteria of appropriateness and prescriptive constraints. The “Gazzetta Ufficiale” is a document containing the laws passed by the government.This action arised a widespread mediatic debate, as the unusual pairing of the prescription appropriateness (“cultural and professional”) and penalty mechanisms (“contractual interest and trade union”) have been considered to be insufficiently shared with medical doctors. Since respiratory allergies (oculorhinitis and bronchial asthma) have an important epidemiological and social health impact, it becomes important to establish a “virtuous” diagnostic flow chart that must be in line with the innovative concepts of appropriateness, and the new findings on diagnostic procedures. Within the physicians (allergists, pulmonologists, pediatricians, general practitioners, etc.) that interface with patients suffering from respiratory allergies, it also becomes important to establish “who does what” and make the harmonious interplay between the various health care providers. Purpose of this article is to make a contribution to improve these dynamics. The fundamental objective in approaching allergic respiratory diseases is that each patient receives the procedures with the best possible outcome and satisfaction based on knowledge available, the least risk of damage following treatment and the lowest consumption of resources (Fig. 1).

## Slow medicine, choosing wisely and allergic respiratory diseases

In the latest few years, the concept of a “slow medicine” approach to clinical problems gained a worldwide interest, being also endorsed by the former British Medical Journal (BMJ) editor in chief, professor Richard Smith, who wrote that “slow medicine —like slow food and slow lovemaking—is the best kind of medicine for the twenty first century” [10].

“Slow medicine”, as a concept, has been coined by Alberto Dolara, an Italian cardiologist that in 2002 invited his colleagues to give the deserved value to the time spent in improving the patient-doctor relationship, implementing a more “human and thoughtful medicine” [11]. However, the underlying concepts were somehow anticipated from some philosophers such as Ivan Illich that, with his “medical nemesis” published in 1974, argued that the medicalization in recent decades of so many of life’s vicissitudes—including birth and death—and the so called “hubris of medicine” frequently caused more harm than good and rendered many people in effect lifelong patients [12].

Into the context of the “slow medicine” international movement, it recently emerged the need to approach one important clinical problem that every physician faces in everyday clinical practice: the mis-use and over-use of medical resources (including useless exams, surgical interventions, medical treatments, screening procedures…) which is well known to lead to increased healthcare costs (without a proportional patients’ benefit) [13] and possible harm to the patients themselves [14]. The immediate consequence of this, was the emerging of the so-called “choosing wisely” campaign (firstly 2012 in USA and then spread in several other countries) [15, 16] with the main goal of identifying the most inappropriate medical procedures for each specialty (those medical procedures that should be deeply discussed with the doctor because can be useless in the diagnostic work-up and even potentially harmful), to protect patients’ interests through a partnership between health professionals and patients and users [17]. In Italy, this campaign took the name of “Doing more does not mean doing better” [18, 19] and it has been coordinated by slow medicine Italy [20] which invited the most relevant Italian scientific societies to list of the five medical procedures with the highest probability of inappropriateness for each specialty (the Italian Society of Allergy, Asthma and Clinical Immunology, SIAAIC as far as the Italian Society of Pediatric Allergy and Immunology, SIAIP adhered to the projects producing their lists of inappropriate procedures [21, 22].

Focusing on respiratory allergic diseases, SIAAIC identified one important inappropriate procedure that was stated as: “Do not diagnose asthma without having performed lung function tests (including bronchodilator test and/or bronchial challenge)” [21]. A similar recommendation was made by the American Academy of Allergy, Asthma and Immunology (AAAAI) into the context of the American choosing wisely campaign (“Don’t diagnose or manage asthma without spirometry”) [23].

Into a recent description of “choosing wisely” campaign promoted by SIAAIC [21], the authors explained that relying only upon asthma-like symptoms (i.e., dyspnea, chest tightness, cough, wheezing) is not sufficient to make a correct diagnosis of asthma, as these symptoms may be due to other causes (i.e., chronic obstructive pulmonary disease (COPD), congestive heart failure, gastroesophageal reflux disease, hyperventilation syndrome, etc. [24–27]. Putting a diagnosis of asthma based only on symptoms can be harmful for patients, as they may be receive a wrong treatment for their complaints. This aspect is even more important when patients suffer from relevant comorbidities, as it happens in the elderly [28]. International asthma guidelines stress the need of performing lung function assessment to identify bronchial hyperreactivity and/or reversibility of bronchial obstruction [29]. Patients with asthma-like symptoms and normal spirometry should underwent a non-specific bronchial challenge (i.e.: with methacholine) while those with an obstructive spirometric pattern should be evaluated for reversibility with a bronchodilating test (i.e., with salbutamol). Beyond the increased costs of care, the consequences of misdiagnosing asthma include delaying a correct diagnosis and treatment [26]. The same Authors suggested also not to perform serological allergy tests (i.e., total IgE, specific IgE, component-resolved diagnosis) as first-line tests or as “screening” of respiratory allergy [21]. This is justified by the fact that cutaneous allergometric tests give faster results, are less invasive and cheaper than serological tests. Moreover, there is a moderate evidence that skin tests have at least the same diagnostic accuracy of serological tests [30, 31]. Exceptions to this recommendation have been identified in situations in which cutaneous tests are not feasible (i.e., hypo- or hyper-reactive cutaneous states), or when an accurate skin prick test extract is not available [32], or if the clinical history suggests an unusually greater risk of anaphylaxis from skin testing [33] (this latest eventuality is very rare in the context of respiratory allergic diseases [34]. As far as total IgE assessment, SIAAIC recommendation is that it is of limited clinical utility in most cases, as it is aspecific and not indicative of allergic sensitization: allergic patients may have both normal or elevated total IgE levels, and patients with high total IgE levels are not necessarily atopic [32–35]. Measuring total IgE is otherwise indicated for the diagnosis of allergic bronchopulmonary aspergillosis, hyper IgE syndrome, as well as inclusion criterion for the correct prescription of anti-IgE therapy in severe allergic asthma [21]. Moreover, SIAAIC experts recommend that all serum allergological tests should be interpreted by specialists/experts in allergy and clinical immunology, since an incorrect interpretation can lead to inappropriate/useless therapeutical approaches, which may be harmful for the patient’s health [21]. Concerning the pediatric aspect (in addition to diagnostic procedures that are similar to adults) SIAIP identified one relevant inappropriate procedure in respiratory allergy work-up: “Do not give mucolytic agents to asthmatic children” [22]. In asthmatic children, in fact, airway inflammation, mucosal edema and mucus hypersecretion worsen bronchial obstruction by the formation of mucous plugs. Studies on mucolytics taken during asthma exacerbations or as add-on treatment of chronic therapy of asthma showed that these drugs are not able to improve asthma outcomes but they can even induce harmful adverse events [36–38].

## Appropriateness, allergists, allergy setting, diagnostic procedures, and allergic respiratory diseases

The incidence and prevalence of bronchial asthma and allergic rhinitis are increasing worldwide. Allergic rhinitis currently affects between 10 and 30 % of the population. Allergic rhinitis is a risk factor for asthma and its comorbidities of allergic rhinitis include: sinusitis, nasal polyposis, conjunctivitis, otitis media with effusion, upper respiratory infections, mouth breathing, and sleep disorders. The prevalence of asthma in different countries varies widely, but the disparity is narrowing due to rising prevalence in low and middle income countries as they are adopting a more westernized lifestyle. In general, patients with asthma are inadequately managed, and asthma and rhinitis are both under-recognized [39]. In addition, there is a large variation among countries in the delivery of care to those suffering from asthma and allergy [40]. What is common among countries, however, is that the majority of patients who seek medical advice for allergy and asthma are seen initially in primary care (or receive advices from pharmacists), because there is an inadequate number of allergists to meet the needs of so many patients [41]. As rightly stated in the World Allergy Organization (WAO) White Book [42]: “the allergist is an expert in tailoring therapy to the individual patient and adjusting treatment dosages in more severe or complex cases. The main defining characteristics of allergists are their appreciation of the importance of external triggers in causing diverse diseases; their expertise in both the diagnosis and treatments of multiple system disorders, including the use of allergen avoidance and the selection of appropriate drug and/or immunological therapies; and their knowledge of allergen specific immunotherapy practices. Misinterpretation of the results of diagnostic tests by nonspecialists can lead to over-diagnosis and inappropriate management which can be harmful for the patient. It may lead to over-prescription of therapy and costly and unnecessary allergen avoidance measures, including exclusion diets that can lead to nutritional deficiency and secondary morbidity. Conversely, the under-appreciation of the severity of asthma can lead to life-endangering under-treatment or the lack of potentially life-altering immunotherapy. The cost-effectiveness of allergist consultation will be demonstrated by improved patient outcomes and experiences, together with a reduction in unnecessary expenditure by payer, society or patient/family”. Therefore, The WAO White Book defines the central role of the Allergist in the management of allergic diseases to ensure an adequate level of health care and “appropriateness*”.*

So, allergic respiratory diseases generate an increase in healthcare-related costs, including secondary costs such as absenteeism and presenteeism at work and at school, if not properly treated. One of the challenges ahead is reconciling the pressing demand of increasingly complex performances with the progressive decrease in the number of allergists. A possible solution can be achieved from the educational role that the allergists should assume with respect to other health professionals, who contribute to the management of allergic diseases. This would result in a different levels-based management probably able to increase the overall efficiency of the system.

In Italy, up to now, there are some critical aspects because Allergology, even though it is recognized as a “precision professional field”; indeed, the basic levels of care (LEA = Livelli Essenziali di Assistenza) as presently defined consider allergy and dermatology as a unique area.

In the recent decree, known as “Appropriateness Decree” of the Italian Ministry of Health, the allergological visit is the first and most important moment before starting the diagnostic work-up. The Appropriateness Decree ratifies that most allergometric tests (i.e., skin prick tests, patch tests, and intracutaneous tests), and some therapeutic procedures, like allergen-specific immunotherapy (AIT), can be performed only after an allergological visit, that is requested after a preliminary evaluation by a general Practitioner or a General Pediatrician. Furthermore, some procedures can be requested only by an allergy specialist: in vivo tests for drugs and hymenoptera, and administration of immunotherapy for hymenoptera venom (VIT).

Finally, some in vitro tests, as specific IgE dosage, are second level tools that must be performed only after in vivo tests or if skin prick test is not conclusive or not feasible. An in vitro test “as a screening” to evaluate respiratory allergy is not recommended. These recommendations are in line with what suggested by SIAAIC in its “choosing wisely” document [21].

Anyway, the conditions to perform in vitro tests depend on the appropriateness of the General Practitioner request. It is also necessary to define the correct number and type of allergens to test. Usually the number of eight allergens is not exceeded, obviously chosen according to the botanic profile of each region. An example of panel of largely present allergens could be: House Dust Mites (Dermatophagoides pteronissinus or farinae), grass (*lolium, dactilis or festuca*), birch, wall pellitory, *alternaria*, cat, dog, cypress or ragweed.

The component resolved diagnosis (CRD) is considered a third level tool. A molecular diagnosis allows to distinguish “false” from “true” sensitization in polisensitized patients. The “WAO ARIA consensus document on molecular based allergy diagnostics”, emphasized its use in particular situations: in polisensitized patients it is used to evaluate if there is a true polisensitization to “genuine molecules” (Phl p 1, Phl p 5, Bet v 1, Amb a 1, Ole e 1, Cup a 1 Par j 2) or cross-reactivity to panallergens such as Profilin or Polcalcine, or to identify the trigger allergens in order to choose the correct immunotherapy [43].

A molecular diagnosis is essential to properly design the composition of allergen specific immunotherapy in patients polisensitized to pollens. Several studies have demonstrated that the use of CRD leads to a relevant change in the prescription of allergen immunotherapy [44].

Moreover, CRD may allows to identify the sensitization patterns which are considered risk factor for severe asthma (i.e., some molds allergens: Alt a 1, Asp f 3, Asp f 4, or dog dander allergen: Can f 5) and the availability of these outcomes could anticipate the development of the disease and therefore identify those patients who will need more strict attention to prevent the onset of a more severe asthma.

More consistent with the interest of national health care services is the ability of CRD to identify food allergens that represent a risk of anaphylaxis or of severe asthma attacks, (asthma associated with food allergy is usually severe or difficult to treat).

Another important aspect is the integrated management of severe asthma. Indeed, approximately 5 % of patients with asthma have a severe, treatment-refractory disease, with a high direct health-care costs (medication burden) and high indirect costs (lost productivity). A recent study reported for the first time the efficacy of a systematic assessment at dedicated severe asthma centers in UK with a significant reduction in health-care use and oral steroid burden [45]. Mean oral steroid dose changed from about 15 mg to about 10 mg and fewer subjects required an oral steroid burst (91 vs 77 %). Hospital admission and primary care or emergency department visits were less, respectively from 48 to 38 % and from 88 to 76 %. It may be of interest for health care services to identify centers able to offer adequate respiratory and allergic diagnostic tools and complete care of the severe asthmatic patient, with a concrete control of the direct costs of therapy (i.e., biological agents or bronchial thermoplasty).

## Conclusions

In Italy (but also in many other countries) there is a severe imbalance between the prevalence of allergic respiratory diseases and the number of hospital and community allergists. This could be improved through the following operational measures: increasing the number of specialists in allergy who each year graduate at the specialty schools; rebalancing the axis of hospital and community care, to develop in parallel both systems and the continuity of care; promoting a real integration between allergists and other physicians (i.e., pneumologists, ENTs, pediatricians, and general practitioners) in the care of the patients with allergic respiratory diseases; developing system solutions to ensure the taking over and the continuity of care of chronic illness and frailty (i.e., severe asthma or elderly patients); overcoming the fragmentation of the territorial organization; and maintaining developing high quality hospital and territorial services through the establishment of clinical audit to verify the appropriateness of diagnostic and therapeutic procedures (Table 1).

**Table 1:** Proposal for a real life distribution of Italian health care resources for respiratory allergic diseases

	I level	II level	III level	Availability, for the National Health Care Service	Availability, for habitants (×1000)
Consultant for allergy	√			TS and HS I level	80–150
Skin prick test (inhalants, foods, latex)	√			TS and HS I level	80–150
Spirometry F/V curve	√			TS and HS I level	80–150
Reversibility test	√			TS and HS I level	80–150
Total IgE		√		HS I level	150–300
Specific IgE, panel for inhalants		√		HS I level	150–300
Specific IgE, panel for foods		√		HS I level	150–300
Component resolved diagnosis			√	HS I–II level	600–1200
Spirometry, lung volumes^a^		√		HS I level	150–300
Spirometry, DLCO^a^		√		HS I level	150–300
Methacholine–challenge^a^		√		HS I level	150–300
Mannitol challenge^a^			√	HS I–II level	300–600
Exhaled nitric oxide (FeNO) measurement		√		HS I level	150–300
Exercise challenge^a^		√		HS I level	150–300
CPET^a^			√	HS I–II level	300–600
Severe asthma center^b^			√	HS I–II level	300–600
HymenopteraVIT center^b^		√		HS I–II level	300–600
Pharmacoallergy center^b^		√		HS II level	600–1200

*TS* territorial services, *HS* hospital setting, *CPET* cardiopulmonary exercise testing, *VIT* venom immunotherapy

^a^Resources to be present in clinical respiratory physiology unit

^b^Board certified centers

To improve the appropriateness, in the field of respiratory allergy, a direct interaction between specialists and policy makers/institutions is mandatory, to better identify the medical needs and the patients’ needs and to properly re-allocate resources.

As a testification of the unmet needs ad of the claim for appropriateness, we convened this cross-sectional task force, which involved different specialists, different scientific societies and a patients’ association. This document is therefore not intended as a guideline, or as a scientific statement, but rather as an approach to claim a more strict cooperation among all the involved health-care operators, including deciders and governmental authorities.

## Abbreviations

EBM: evidence-based medicine
BMJ: British Medical Journal
SIAAIC: Italian Society of Allergy, Asthma and Clinical Immunology
SIAIP: Italian Society of Pediatric Allergy and Immunology
AAAAI: American Academy of Allergy, Asthma and Immunology
COPD: chronic obstructive pulmonary disease
WAO: World Allergy Organization
AIT: allergen-specific immunotherapy
VIT: venom immunotherapy
CRD: component resolved diagnosis
